# Predictors of COVID-19 Vaccine Acceptance and Hesitancy among Healthcare Workers in Southern California: Not Just “Anti” vs. “Pro” Vaccine

**DOI:** 10.3390/vaccines9121428

**Published:** 2021-12-02

**Authors:** Alex Dubov, Brian J. Distelberg, Jacinda C. Abdul-Mutakabbir, W. Lawrence Beeson, Lawrence K. Loo, Susanne B. Montgomery, Udochukwu E. Oyoyo, Pranjal Patel, Bridgette Peteet, Steven Shoptaw, Shahriyar Tavakoli, Ara A. Chrissian

**Affiliations:** 1 School of Behavioral Health, Loma Linda University, Loma Linda, CA 92350, USA; bdistelberg@llu.edu (B.J.D.); smontgomery@llu.edu (S.B.M.); bpeteet@llu.edu (B.P.); 2 School of Pharmacy, Loma Linda University, Loma Linda, CA 92350, USA; JAbdulmutakabbir@llu.edu; 3 School of Public Health, Loma Linda University Loma Linda, CA 92350, USA; lbeeson@llu.edu; 4 School of Medicine, Loma Linda University, Loma Linda, CA 92350, USA; lkloo@llu.edu (L.K.L.); PRAPatel@llu.edu (P.P.); STavakoli@llu.edu (S.T.); AChrissian@llu.edu (A.A.C.); 5 School of Dentistry, Loma Linda University, Loma Linda, CA 92350, USA; UOyoyo@llu.edu; 6 Department of Family Medicine, University of California Los Angeles, Los Angeles, CA 90032, USA; SShoptaw@mednet.ucla.edu

**Keywords:** COVID-19 pandemic, SARS-CoV-2, COVID-19 vaccine, vaccine hesitancy, vaccine acceptance, healthcare professionals

## Abstract

In this study, we evaluated the status of and attitudes toward COVID-19 vaccination of healthcare workers in two major hospital systems (academic and private) in Southern California. Responses were collected via an anonymous and voluntary survey from a total of 2491 participants, including nurses, physicians, other allied health professionals, and administrators. Among the 2491 participants that had been offered the vaccine at the time of the study, 2103 (84%) were vaccinated. The bulk of the participants were middle-aged college-educated White (73%), non-Hispanic women (77%), and nursing was the most represented medical occupation (35%). Political affiliation, education level, and income were shown to be significant factors associated with vaccination status. Our data suggest that the current allocation of healthcare workers into dichotomous groups such as “anti-vaccine vs. pro-vaccine” may be inadequate in accurately tailoring vaccine uptake interventions. We found that healthcare workers that have yet to receive the COVID-19 vaccine likely belong to one of four categories: the misinformed, the undecided, the uninformed, or the unconcerned. This diversity in vaccine hesitancy among healthcare workers highlights the importance of targeted intervention to increase vaccine confidence. Regardless of governmental vaccine mandates, addressing the root causes contributing to vaccine hesitancy continues to be of utmost importance.

## 1. Introduction

COVID-19, caused by infection with severe acute respiratory syndrome coronavirus 2 (SARS-CoV-2), emerged in late 2019 and reached a global pandemic level by March 2020 [1]. By October 2021, 244 million known infections were recorded worldwide, with 45 million cases in the United States (U.S.) resulting in over 736,000 deaths [2]. Unprecedented global research efforts produced effective COVID-19 vaccines in record time, with the first doses becoming available in December 2020 [3]. Healthcare workers (HCWs) were the first group in the U.S. to be offered COVID-19 vaccinations. However, several months into the vaccination effort, many remain hesitant and unvaccinated despite increasingly stringent vaccination policies.

Vaccine hesitancy, the leading threat to global health [4], is the refusal of vaccination despite availability and accessibility. HCW vaccine hesitancy can be rooted in many factors including fears about safety and efficacy [5,6], preference for physiological herd immunity (i.e., natural inoculation) [7], distrust in government [8,9], maintaining a sense of personal freedom [6,10], sociodemographic characteristics, and broader external or organizational factors [11]. A recent scoping review of 35 studies published after vaccine authorizations found a hesitancy rate of 22.5% among 76,471 HCWs [12]. Hesitancy rates among HCWs are occupation and context dependent. For instance, 96% of the practicing physicians in that study had been fully vaccinated [13]. In contrast, only about a third (37.5%) of HCWs in skilled nursing facilities had been vaccinated, compared to three-fourths (77.8%) of nursing home residents [14]. In another study, data collected from 2500 U.S. hospitals show regional differences in HCWs vaccination rates—from a high of 99% at Houston Methodist Hospital, the first hospital to introduce vaccination mandates, to a low of between 30% and 40% at Florida hospitals [15]. No identified studies have been conducted in the Southern California region. 

Experts suggest that given our global connectivity, addressing the threat of COVID-19 is highly dependent on increasing vaccination rates everywhere to reach herd immunity levels [16]. HCWs are a critical partner in moving vaccine-hesitant populations toward vaccination. HCWs are more trusted and viewed more positively than elected officials or government agencies [17]. Therefore, addressing COVID-19 vaccine hesitancy among HCWs is a complex but important task in reaching herd immunity. As hesitancy is not uniform, vaccine uptake and preference analyses will allow us to detect HCW subgroups with low vaccination acceptance. Identifying the determinants of vaccine hesitancy among these subgroups and then tailoring the vaccination campaign to fit each sub-group’s concern is essential to addressing vaccine hesitancy. 

In the present study, we sought to determine what proportions of HCWs in Southern California were accepting of, hesitant about, or resistant to a COVID-19 vaccine. Additionally, we sought to profile HCWs who are hesitant about or resistant to a COVID-19 vaccine by identifying key sociodemographic, occupational, political factors and specific beliefs that distinguish them from those who accept a COVID-19 vaccine. While this cross-sectional exploratory study had no explicit hypotheses, our approach was guided by several assumptions: (1) most HCWs would accept vaccination, (2) HCWs job characteristics including direct patient interaction would facilitate vaccine acceptance, (3) sociodemographic characteristics of HCWs such as gender, race, age, and educational attainment will be associated with intention to take vaccine, (4) vaccine hesitancy among HCWs will be influenced by several factors, including insufficient knowledge about the vaccines, misinformation from social media, political affiliation, and previous COVID-19 infection. Understanding the characteristics that predict vaccine hesitancy among HCW subgroups will enable health administrators to apply and evaluate tailored interventions.

## 2. Materials and Methods

We conducted an online, cross-sectional survey of HCWs at two large hospital systems (academic and private) in Southern California. The study protocol was reviewed by the respective IRBs of each participating institution and deemed exempt. Both hospitals started vaccinating their HCWs against COVID-19 on 17 December 2020. 

### 2.1. Sample and Recruitment Strategy

Recruitment occurred relatively early after vaccination distribution; between 5 and 26 February 2021 at the academic hospital, and 3 and 17 April 2021 at the private hospital. We distributed the online Qualtrics survey via institution-wide email listservs, with 8848 recipients at the academic hospital and 3062 recipients at the private hospital. Listserv recipients included physicians, nurses, advanced practice providers, pharmacists, other allied health professionals, administrators, and nonclinical ancillary staff. All employees of both hospital systems were invited to participate in the study. The initial invitation email to complete the survey was followed by two email reminders to encourage participation. The survey was anonymous and voluntary. 

### 2.2. Data Collection Process

A working group developed a survey to understand HCWs’ knowledge, attitudes, and perceptions about the COVID-19 vaccination. The survey was based on previous questionnaires conducted in the context of the 2009 H1N1 flu pandemic. It was pilot-tested with 7 healthcare professionals and revised to ensure readability and understandability. The final survey included exclusively forced-choice questions to avoid missing data. The overall survey response rate was 20.9% (2491 respondents/11,910 total possible recipients) with a dropout rate of 17%. This modest response rate can be attributed to the spike in COVID-19 infections and hospitalizations in Southern California, coinciding with the dates when the study survey was administered. The increased workload and burnout among HCWs presented a barrier to survey completion.

### 2.3. Measures

The survey instrument was composed of five parts: (1) Demographics: including age, gender, race, ethnicity, education level, self-reported history of chronic illness, income level, household size, and political party affiliation; (2) Clinical characteristics: including position within the healthcare field, clinical work setting, medical specialty, frequency of contact with COVID-19 patients, and self-reported history of flu vaccination; (3) COVID-19-related misinformation: including belief in a synthetic origin of the virus, belief in COVID-19 being a hoax, and belief that COVID-19’s impact on the healthcare system is exaggerated; (4) COVID-19 knowledge: understanding that COVID-19 is more deadly and contagious than seasonal flu, estimated COVID-19 mortality for self and an average American, understanding of COVID-19 vaccine effectiveness; (5) COVID-19’s impact: COVID-19’s financial impact and whether someone close had a severe illness or died due to COVID-19. We derived the primary outcome, COVID-19 vaccine behavior/hesitancy, from two questions—(a) receipt of any dose of a COVID-19 vaccine and (b) in case of a negative answer, intention to receive a COVID-19 vaccine within the next six months. We considered answers “unsure”, “probably not”, and “definitely not” indicative of COVID-19 vaccine hesitancy. The 37-item survey took, on average, 15 min to complete.

### 2.4. Analysis

All data were exported into SPSS 26.0 for analysis. Data were first reviewed at a univariate and descriptive statistics level, and the ability of the data to conform to the assumptions of the planned analysis [18]. Analyses included a review of the vaccination rates and presentation of the descriptive statistics. We then performed multinomial logistic regression. This method is similar to logistic regression but allows for an outcome variable with 3 or more levels since our outcome variable had three categories (vaccinated, not vaccinated, and hesitant). The data supported assumptions for multinomial logistic regression. The outcome variable was structured to have three categories: (1) Vaccinated (2) Not vaccinated, and (3) Hesitant to be vaccinated. Independent variables were fit to the multinomial logistic regression model in hierarchical order with demographic variables added first, followed by participant’s occupation and clinical area of employment. Next, we entered COVID-19 and vaccine-related variables, sources of news, political party affiliation, and frequency of patient contact. The independent variables were fit as covariates. Categorical or nominal variables were dummy coded prior to estimation.

Realizing that there is a continuum between total acceptance and complete refusal of vaccinations, we conducted clustering analysis to further describe groups of currently unvaccinated HCWs holding varying degrees of indecision about vaccination. We used the kernel k-means method (Kernlab R package) and the kernel function of the radial basis (Gaussian kernel) to perform a cluster analysis. This method, representing a more generalized k-means approach to cluster analysis, is well-suited for linear and nonlinear separable inputs because the data type is usually unknown. Kernel k-means cannot determine the number of clusters. Therefore, we used the variance ratio criterion (VRC) to determine the number of clusters. VRC was selected due to its excellent performance against other internal criteria to determine the number of clusters [19]. The literature suggests that VRC is the most effective criterion for purposes of cluster number determination [20,21]. We set the number of clusters to four, with the highest value based on the variance ratio criterion (Table 1). 

## 3. Results

### 3.1. Sample Characteristics 

Overall, 2491 respondents answered the survey between 5 February and 17 April 2021. Table 1 presents the descriptive characteristics of the sample. Among 2491 HCW respondents, 35% were nurses, 19% were physicians, and 7% were administrators. Respiratory therapists, advanced practice providers, and pharmacists each represented about 3% of the sample. The remaining 29% were other allied health professionals. The majority of participants were White (73%), non-Hispanic (77%), women (75%), born after 1965 (75%), and college-educated or higher (74%). The most reported political affiliation was Democrat/leaned Democrat (46%), while 30% were Republican/leaned Republican, and 24% reported no lean to either political party.

The HCWs participating in the survey were significantly impacted by the pandemic. Sixty-one percent reported having at least intermittent contact with COVID-19 patients, with 28% having frequent contact. Thirteen percent reported being diagnosed with COVID-19, and 42% said that someone close to them had suffered severe disability or died from COVID-19. Forty-seven percent of participants stated that the pandemic had negatively affected them financially. 

There was diversity in beliefs about the virus and the vaccine. Twenty-three percent considered seasonal influenza as more contagious than COVID-19, 32.6% overestimated the mortality associated with COVID-19, while 11.5% underestimated its severity. A sizeable fraction of the sample held conspiratorial beliefs about COVID-19, with 38% of the sample believing the virus is or could be manmade, 15% suggesting the impact of COVID-19 is overblown, and 6% not rejecting the notion that the pandemic is a hoax. A large majority (80.2%) identified vaccine efficacy to be 90% or greater.

COVID-19 vaccination rate at the time of survey administration was high, with 2103 (84.4%) reporting having received it. Vaccine uptake was highest among physicians (96.2%) and lowest among respiratory therapists (70.3%), while 78.6% of nurses were vaccinated. Among the 391 unvaccinated HCWs at the time of the survey, 87 (3.5%) were willing to receive the vaccine, leaving 304 HCW (12.2%) whom we classified as vaccine hesitant. Additional sample characteristics can be found in Table 2. 

### 3.2. Predictors of Vaccination Intentions

We used a Chi-square test to determine the likelihood of vaccination for all participants. The model’s overall fit was excellent (Likelihood Ratio Chi-sq = 975.8, df = 96, *p* < 0.001) and achieved an overall correct classification rate of 88.6%. The Cox and Snell R2 was moderate at 0.33. This reflects the overall characteristics of the model in that the model was strong in predicting whether a participant was vaccinated (correct classification = 97.9%) but had a lower success rate at predicting the not vaccinated group (correct classification = 43.3%) and was fairly weak in predicting the hesitant group (correct classification = 26.6%). Thus, our model effectively predicted the likelihood of a participant being vaccinated. Table 3 presents the univariate distribution of data across the three outcome groups (vaccinated, not vaccinated, and hesitant). 

While the overall fit was strong, only certain variables offered explanatory power (Table 4).

Asian American participants were highly likely to be vaccinated, and to a lesser degree, younger HCWs. No other demographic variables added predictive value to the outcome. In the remainder of the model, the most significant predictor of COVID-19 vaccination was the individual’s approach to influenza vaccines: those with recent or previous flu vaccinations were more likely to have received the COVID-19 vaccine. Furthermore, HCWs working in an outpatient area of the health systems were more likely to be vaccinated, as were those leaning Democrat. Conversely, the most significant predictors of a participant not getting vaccinated included: inaccurate knowledge of COVID vaccine efficacy, belief that COVID-19 is a manmade virus, belief that the impact of COVID-19 is exaggerated, perceived low risk of dying if infected, having a prior diagnosis of COVID-19, and being financially impacted by COVID-19.

The comparison of “vaccinated versus hesitant” showed several differences. Overall, older, higher educated participants who lived in homes with more family members were more likely to be hesitant to receive the vaccine. Conversely, physicians and HCWs with higher income were less likely to be hesitant. Important variables that did not predict either HCW vaccination or hesitancy included gender, presence of chronic illness, specialty area of practice, source of news, frequency of contact with COVID-19 patients, and having someone close affected by COVID-19.

### 3.3. K-Means Cluster Analysis 

To determine characteristic groupings of the unvaccinated, we conducted a K-means cluster analysis. According to the values of the variance ratio criterion, participants were separated into four clusters (Table 5). 

**Figure 1:**
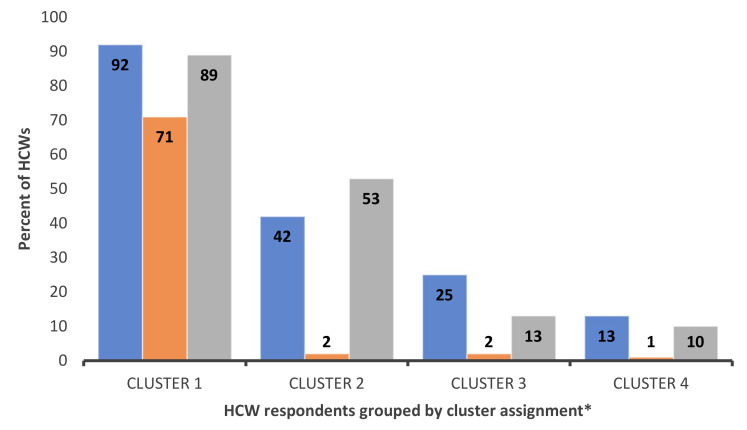
HCW support of COVID-19 conspiracy theories by cluster. Blue: HCWs that believe COVID-19 is a manmade virus; Orange: HCWs that believe COVID-19 is a hoax; Grey: HCWs that believe the impact of COVID-19 is exaggerated; * Cluster derivation and definitions are provided in surrounding text.

Respondents grouped in cluster 1 “misinformed” (*n* = 38) were slightly older and leaned Republican. They strongly opposed the COVID-19 vaccine, refusing to receive and/or recommend the COVID-19 vaccine. This group underestimated both the COVID-19 vaccine’s efficacy and COVID-19 mortality. They consider seasonal flu as more contagious and deadly than the COVID-19 virus. Finally, members of this cluster were more likely to believe several COVID-19 conspiracies (e.g., COVID-19 is a hoax). As seen in Figure 1, members of this group are subjects of disinformation from politically leaning news media. 

Members of ***cluster 2 “uninformed”*** (*n* = 94) tended to be less educated (60% lacking undergraduate degree), were more likely Hispanic/Latinx (47%), and worked in outpatient areas (33%) as allied health providers (60%). This cluster is the second least willing to receive the COVID-19 vaccine. This group underestimated the impact of the pandemic and the efficacy of the vaccine. They were primarily unsure about comparisons between COVID-19 and seasonal influenza. Unlike members of cluster 1, this group is less impacted by disinformation but lacks access to reliable and easy-to-understand vaccine information.

***Cluster 3 “undecided”*** (*n* = 86) members were more open to receiving the COVID-19 vaccine, with half of the respondents unsure about vaccine receipt. Members of this cluster were predominantly White nurses and respiratory therapists working in an ICU. They understood the personal risk of exposure to the virus and knew the severity of COVID-19 disease, correctly assuming it is deadlier than seasonal flu. Participants in this cluster strongly leaned Republican. 

**Figure 2:**
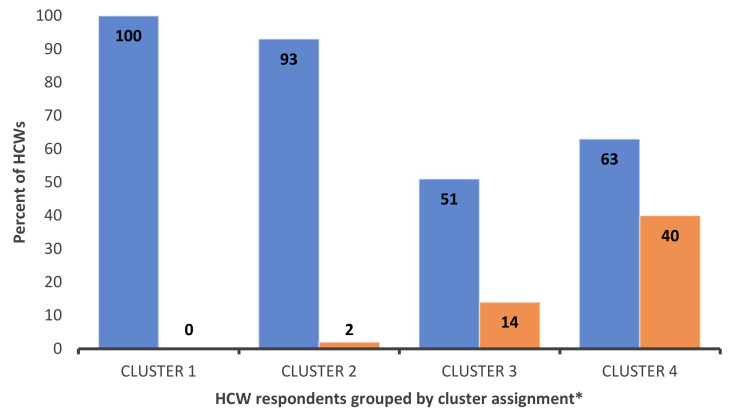
HCWs willingness to receive COVID-19 vaccine versus willingness to recommend vaccine to others. Blue: HCWs that probably or definitely would NOT receive COVID-19 vaccine; Orange: HCWs that probably or definitely WOULD recommend COVID-19 vaccine to others; * Cluster derivation and definitions are provided in surrounding text.

***Cluster 4 “unconcerned”*** (*n* = 86) members were younger and racially diverse. This cluster is the most educated and leaned Democrat. Members of this cluster had an accurate knowledge of the vaccine’s efficacy and the lowest support of COVID-19 conspiracies. While hesitating to receive the vaccine themselves, respondents in this cluster were willing to recommend it to others (Figure 2). 

## 4. Discussion

We found that HCWs are a heterogeneous group with varying attitudes toward vaccination. In our cohort of 2491 HCWs who had been offered the vaccine and responded to our survey, 2109 (84%) were vaccinated, and 304 (12%) were vaccine-hesitant. Vaccination and hesitancy rates varied by age, ethnicity, professional roles, work setting, political affiliation, attitudes toward influenza vaccination, and knowledge of both COVID-19 severity and vaccine efficacy. Furthermore, HCWs who believe that the media has exaggerated the severity of the pandemic perceived the risk of vaccination to be greater than the risk of infection. Our findings parallel those of other studies [8,22,23,24,25] and underscore the importance of tailored communication strategies to disseminate scientific data to increase HCWs’ confidence in the COVID-19 vaccine. 

We found that vaccine hesitancy was associated with older age and higher education. In additional analyses, age and education positively correlated with political affiliation (Republican) and occupation (nurse), respectively. Highly educated nurses were more hesitant to accept vaccination, often citing concerns in open-ended comments over unrealized side-effects of the vaccination, including its potential impact on fertility and pregnancy. HCWs who were leaning Republican tended to be older and more hesitant of COVID-19 vaccination. This underscores the politicized nature of the pandemic and the potential of one’s political affiliation to have a more substantial influence on vaccination decisions than age and susceptibility to the virus [6]. This finding is in line with surveys of the general public [26,27,28]. Prior COVID diagnosis was also associated with vaccine hesitancy, potentially reflecting HCWs’ preference for physiological immunity. Finally, as in other studies [29,30,31], one’s family size was predictive of vaccine hesitancy. This finding can be explained through interrelated factors, including socioeconomic status.

Trust is the essential factor in gaining acceptance of the COVID-19 vaccine. Alongside the public, HCWs have been exposed to conspiracy theories such as claims that the government intentionally created COVID-19 or that health organizations have exaggerated its lethality for financial or political purposes. These conspiratorial beliefs were strongly associated with vaccine refusal and were not limited to HCWs with lower education. Cognitive biases can be an underlying cause of conspiratorial beliefs even among educated HCWs. For instance, the availability heuristic may skew their perception of vaccination safety, while confirmation bias may strengthen their vaccine hesitancy through selective exposure to evidence [32,33]. HCWs financially impacted by the COVID-19 pandemic were likely to exhibit vaccine skepticism. This finding may point to the difference between COVID-19 disinformation among White and well-educated HCWs and inequality-driven medical mistrust among racially diverse groups of HCWs made vulnerable by the pandemic. 

We found four distinct clusters among vaccine-hesitant HCWs, suggesting that the dichotomous “anti-vaccine vs. pro-vaccine” separation of HCWs may not be adequate in informing interventions. 

***Cluster 1 members (misinformed)*** are dominated by vaccine-related myths and skeptical attitudes toward vaccine effectiveness. This cluster is the highest on the vaccine hesitancy continuum but also the smallest. Building trust within this group may be challenging and require strategies that utilize direct peer-to-peer communication [34]. For instance, HCWs may become “vaccine ambassadors” by directly engaging their colleagues in common settings (e.g., social media groups) and addressing relevant misinformation, as modeled by the “Nurses Who Vaccinate” organization members. However, directly reacting to misinformation may produce backlash among members of this cluster [35]. A stronger approach is to adopt methods used by the anti-vaccination movement, relying on personal and emotional narratives [36]. These narratives may center on “conversion” of an anti-vaccination HCW to pro-vaccine ideology [37] or stories highlighting personal risks of COVID-19 that can be avoided through vaccination [38]. Vaccine mandates may also be effective in increasing uptake among this group. However, mandates carry the risk of completely isolating this group and losing them to the profession at a time with an already high dropout due to COVID-19 and other burn-out [39]. 

***Cluster 2 (uninformed)*** appears to be the sub-group of HCWs with the greatest need for accurate and easy-to-understand vaccine information. An educational campaign providing evidence-based information on the safety and effectiveness of the vaccination, with contents addressing their concerns, could further COVID-19 vaccine acceptance in this group [40]. Such an educational campaign needs to employ various communication channels, including printed materials, email blasts, social media, and short videos. This group seems to be negatively affected by the changing and evolving information around COVID-19. Clearer messaging about the reasons and need for evolving recommendations are essential for this group. This messaging can be achieved by hosting open discussions where HCWs at different levels can provide input and ask questions. 

***Members of cluster 3 (undecided)*** are the closest to acceptance on the vaccine hesitancy continuum. Their hesitancy may be attributed to partisan group identity. Several communication strategies can be effective in reaching this group. One is to highlight the non-partisan nature of vaccination decisions and endorsement of the COVID-19 vaccine from various political figures [41]. It is important to emphasize that vaccination is a social contract in which cooperation is the morally correct choice [42]. Leveraging social norm cues is another tactic to increase vaccination in this group. Several studies have documented the impact of perceived vaccine coverage in the social circle on vaccination behavior for influenza [43] and HPV [44]. Additionally, members of this group might be more inclined to accept vaccination resulting from a personal choice rather than coercion. Motivational interviewing is an effective approach to support a sense of personal freedom while decreasing vaccine hesitancy. Both CDC [45] and WHO [46] have released training modules describing this technique. 

Finally, ***cluster 4 (unconcerned)*** are willing to recommend vaccination to others but have not been vaccinated themselves (yet). Their hesitancy may stem from under-estimating personal risks. Interventions rooted in behavioral economics (nudges) may increase vaccination rates in this group [47]. For instance, some hospitals use peer pressure to encourage vaccination (e.g., HCWs wearing “I am vaccinated” badges, public posting of vaccination rates) [48]. Pre-scheduling vaccination appointments or providing vaccination bonuses are promising evidence-based nudges to reach this group [49]. Additionally, messaging promoting prosocial motivations (e.g., protecting one’s community from COVID-19) can enhance vaccination intentions in this group [50].

### Limitations

While our study has many strengths, including recruitment from both a public and private hospital system, and across a broad range of HCWs, in this constantly evolving response to the pandemic, it is limited by its “point in time”, to a time when vaccinations were fairly new. Our response rate of 20.9% introduces nonresponse bias and may not be fully representative of the HCWs population at the two hospital systems or generalized to other hospital systems. However, this response rate mirrors other surveys on the same topic systematically reviewed by Li et al. [11]. As there is no scientifically proven lower limit for an accepted survey response rate, several approaches, such as early- to late-responder comparisons, may help address nonresponse bias. For our study, there were no significant differences between the early and late responders. Additionally, our study is cross-sectional and does not allow us to establish temporal causality, explore vaccination uptake and relies on self-report. It is likely that HCWs’ opinions on vaccination evolved over time. Hence, future surveys using validated instruments and relying on vaccination rates are needed to capture these changes. In this survey, anonymity was stressed, and the pressure to be vaccinated had not yet been a public discussion. This likely resulted in important information that later may have been harder to obtain. Our results remain highly relevant, even when California legislation now calls for mandated HCW vaccination. Vaccination mandates have minimal influence on vaccine hesitancy. According to several recent surveys [51], about 50% of vaccine-hesitant HCWs would quit, start looking for other employment or both if their hospital system introduced a mandate. Our data point to important subgroups that need to be engaged as it is HCWs’ advice that will help sway public uptake of vaccination. Our clusters, each in their own way, will need to be convinced of the “why” as they play an important role in reaching similarly minded groups of vaccine-hesitant communities that are often in close contact (echo chambers).

## 5. Conclusions

HCWs have a strong influence on patient and public perceptions of the COVID-19 vaccines, and therefore are one of the most valuable assets in disease prevention. Unvaccinated HCWs are less likely to recommend the vaccine to others [6,8]. Vaccine hesitancy is the most significant barrier to achieving herd immunity, thus contributing to lingering infection and mortality within strained healthcare systems [4,52]. Our study found diversity in vaccine hesitancy among HCWs and highlights the need for unique and targeted interventions depending on degrees, types, and causes of hesitancy. Consequently, messaging should be tailored to specific subgroups to increase the understanding of the science behind vaccines. Interventions should elicit HCWs’ concerns with empathy, and policymaking should be inclusive of vaccine-hesitant subgroups.

## Figures and Tables

**Table 1:** The number of clusters analyzed by variance ratio criterion.

Number of Clusters	3	4	5	6	7
**Variance ratio value**	2.8167	4.7548	4.2031	3.8492	2.9618

**Table 2:** Demographic characteristics.

	N (%)
**Gender**	
Male	618 (24.81)
Female	1.867 (74.95)
Other/non-binary	6 (0.24)
**Age**	
1946–1964	615 (24.94)
1965–1980	800 (32.44)
1981–1996	998 (40.47)
After 1996	53 (2.15)
**Race**	
White	1.815 (72.86)
Black or African American	123 (4.94)
Asian American	438 (17.58)
Pacific Islander	47 (1.89)
Native American	68 (2.73)
**Ethnicity**	
Hispanic/Latinx	570 (22.88)
Non-Hispanic/Latinx	1.921 (77.12)
**Education**	
Some college	326 (13.09)
Associate degree	319 (12.18)
Bachelor’s degree	823 (33.05)
Graduate degree	397 (15.94)
Doctoral degree	525 (25.10)
**Household income level**	
Less than USD 50,000	124 (4.98)
USD 50,000–100,000	526 (21.12)
USD 101,000–150,000	624 (25.05)
USD 150,000–200,000	405 (16.26)
USD 201,000–250,000	261 (10.48)
Greater than USD 250,000	365 (14.65)
Decline to respond	186 (7.47)
**Political Affiliation**	
Democrat/lean Democrat	1.158 (46.49)
Republican/lean Republican	743 (29.83)
No lean	590 (23.69)
**Occupation**	
Physician	473 (18.99)
Attending	348 (13.97)
Resident	108 (4.34)
Fellow	17 (0.68)
Nurse	869 (34.89)
Nurse practitioner/Physician Assistant	83 (3.33)
Pharmacist	61 (2.45)
Respiratory Therapist	91 (3.65)
Administrator	176 (7.07)
Patient care assistant	738 (29.63)
**Clinical area**	
ICU	604 (24.25)
Non-ICU	853 (34.24)
Emergency Department	177 (7.11)
Outpatient	808 (32.44)
**Clinical Specialty**	
Critical care	187 (7.51)
Adult	104 (4.18)
Pediatric	83 (3.33)
General Medicine	486 (19.51)
Adult	375 (15.05)
Pediatric	111 (4.46)
Subspecialty	975 (39.14)
Adult	744 (29.87)
Pediatric	231 (9.27)
Surgery	310 (12.44)
Emergency	163 (6.54)
Not medical	370 (14.85)
**Contact with COVID-19 patients**	
Frequent *	698 (28.02)
Intermittent **	838 (33.64)
No contact	955 (38.34)
**Recent flu vaccination**	
Yes	2.285 (91.73)
No	206 (8.27)

* direct care once a week/contact with many during one shift, ** direct care less than once a week/consult on the cases.

**Table 3:** Univariate distribution of data across three outcome groups.

	Vaccinated		Hesitant		Not Vaccinated	
	Mean or %	SE	Mean or %	SE	Mean or %	SE
Male	91.10%		6.10%		2.80%	
Latinx	83.20%		11.40%		5.40%	
Black	74.80%		17.90%		7.30%	
Asian	93.20%		5.70%		1.10%	
Age						
<25 years	77.40%		20.80%		1.90%	
25–40 years	81.90%		11.90%		6.20%	
41–55 years	87.00%		8.90%		4.20%	
56–75 years	91.50%		4.60%		3.90%	
Education Level						
Some College	85.30%		8.60%		6.10%	
Associate Degree	80.60%		12.20%		7.20%	
Bachelor’s Degree	81.90%		12.40%		5.70%	
Graduate Degree	85.90%		9.60%		4.50%	
Doctorate Degree	94.40%		3.70%		1.90%	
Chronic Illness	87.80%		8.10%		4.10%	
Household Size	2.94	0.03	3.31	0.08	3.33	0.12
Income						
<USD 50,000	86.30%		9.70%		4.00%	
USD 50,000–100,000	81.60%		12.70%		5.70%	
USD 101,000–150,000	84.00%		11.10%		5.00%	
USD 151,000–200,000	86.40%		9.10%		4.40%	
USD 201,000–250,000	87.40%		8.00%		4.60%	
>USD 250,000	93.40%		2.50%		4.10%	
Occupation						
Nurse	80.80%		7.60%		3.60%	
Physician	96.60%		2.10%		1.30%	
NP/PA	94.00%		3.60%		2.40%	
Administration	93.20%		5.70%		1.10%	
Clinical area						
Intensive Care Unit	82.00%		11.80%		6.30%	
Emergency Department	84.70%		11.30%		4.00%	
Outpatient	90.60%		5.80%		3.60%	
Specialty area						
Adult Critical Care	80.80%		12.50%		6.70%	
Adult Specialty Care	86.60%		8.60%		4.80%	
Peds Critical Care	80.70%		9.60%		9.60%	
Peds Specialty Care	80.50%		13.00%		6.50%	
COVID conspiracies						
COVID is manmade	4.02	0.03	3	0.09	2.65	0.13
COVID is a hoax	4.85	0.02	4.53	0.06	4.16	0.12
COVID impact is exaggerated	4.61	0.02	3.84	0.09	2.99	0.13
COVID vs. Flu						
Flu is more contagious	2.69	0.03	2.90	0.07	3.00	0.11
History of Flu vaccine	88.90%		7.60%		3.50%	
Recent Flu vaccine	89.70%		7.50%		2.80%	
COVID impact						
Financial impact	82.90%		10.90%		6.20%	
Someone close had COVID	83.00%		10.30%		6.70%	
Someone close was hospitalized	85.30%		9.90%		4.80%	
Someone close died	88.60%		8.20%		3.10%	
Estimated COVID mortality						
Underestimate	71.10%		14.60%		14.30%	
Overestimate	89.50%		8.00%		2.50%	
Likelihood of dying from COVID						
High	73.40%		15.70%		11.00%	
Low	93.40%		5.10%		1.60%	
COVID vaccine knowledge						
Underestimate efficacy	58.10%		25.10%		16.80%	
Prior COVID diagnosis						
Recovered from COVID	71.60%		20.20%		8.30%	
Contact with COVID patients						
Frequent	84.10%		10.70%		5.20%	
Intermittent	85.60%		9.70%		4.77%	
No contact	87.60%		7.75%		4.61%	
Political party affiliation						
Democratic	93.90%		4.50%		1.60%	
Republican	78.60%		13.20%		8.20%	
Social media use						
Well connected	3.86	0.02	3.73	0.07	3.83	0.1
News sources						
Cable news	87.90%		8.00%		4.10%	
Mainstream news	91.10%		5.70%		3.20%	
Social media	85.70%		10.20%		4.10%	
Family or friends	73.00%		19.70%		7.40%	

**Table 4:** Predictors of vaccination intention.

	Not Vaccinated			Hesitant		
	aOR [95% CI]	B	*p*-Value	aOR [95% CI]	B	*p*-Value
**Demographics**						
Male	0.66 [0.32,1.37]	−0.41	0.240	0.91 [0.57,1.45]	−0.09	0.701
Latinx	0.58 [0.30,1.10]	−0.55	0.100	0.75 [0.49,1.12]	0.75	0.194
Black	1.07 [0.42,2.71]	0.07	0.740	1.6 [0.85,3.02]	1.6	0.149
Asian	0.10 [0.03,0.31]	−2.28	**<0.001**	0.44 [0.25,0.75]	0.44	**<0.001**
Age	1.55 [1.08,2.22]	0.43	**0.010**	1.83 [1.42,2.36]	1.83	**<0.001**
Education level	1.02 [0.77,1.36]	0.02	0.810	1.33 [1.11,1.59]	1.33	**<0.001**
Chronic illness	1.60 [0.89,2.85]	0.47	0.120	1.44 [0.98,2.14]	1.44	0.070
Household size	1.17 [0.95,1.44]	0.16	0.100	1.19 [1.04,1.37]	1.19	**0.013**
Income	1.04 [0.89,1.22]	0.04	0.590	0.89 [0.80,0.99]	0.89	**0.045**
**Occupation**						
Nurse	1.54 [0.85,2.70]	0.43	0.150	1.03 [0.70,1.54]	0.03	0.867
Physician	1.14 [0.31,4.15]	0.13	0.810	0.29 [0.12,0.67]	−1.25	**0.004**
NP/PA	0.78 [0.12,4.82]	−0.26	0.730	0.3 [0.08,1.14]	−1.21	0.077
Administration	0.35 [0.07,1.81]	−1.06	0.170	0.65 [0.30,1.44]	−0.43	0.287
**Clinical area**						
Intensive Care Unit	0.87 [0.42,1.80]	−0.14	0.600	0.92 [0.57,1.48]	−0.09	0.725
Emergency Department	1.11 [0.19,6.50]	0.1	0.830	0.63 [0.20,2.04]	−0.46	0.442
Outpatient	0.42 [0.21,0.83]	−0.87	**0.009**	0.5 [0.31,0.79]	−0.70	**<0.001**
**Specialty area**						
Adult Critical Care	0.73 [0.21,2.50]	−0.32	0.610	0.78 [0.34,1.81]	−0.24	0.569
Adult Specialty Care	0.98 [0.52,1.85]	−0.02	0.960	1 [0.65,1.55]	0.01	0.976
Peds Critical Care	1.21 [0.34,4.37]	0.19	0.770	0.76 [0.29,1.98]	−0.28	0.570
Peds Specialty Care	0.92 [0.37,2.33]	−0.08	0.870	1.18 [0.64,2.17]	0.17	0.591
**COVID conspiracies**						
COVID is manmade	1.37 [1.12,1.68]	0.32	**0.002**	[1.19,1.55]	0.31	<0.001
COVID is a hoax	0.82 [0.62,1.10]	−0.19	0.195	[0.68,1.10]	−0.14	0.235
COVID impact is exaggerated	1.66 [1.33,2.01]	0.51	**<0.001**	[1.01,1.41]	0.17	**0.043**
**COVID vs. Flu**						
Flu is more contagious	0.68 [0.52,0.89]	−0.40	**0.005**	0.91 [0.76,1.08]	−0.10	0.261
History of Flu vaccine	0.47 [0.23,0.93]	−0.77	**0.032**	0.33 [0.20,0.55]	−1.12	**<0.001**
Recent Flu vaccine	0.09 [0.04,0.17]	−2.47	**<0.001**	0.29 [0.17,0.50]	−1.23	**<0.001**
**COVID impact**						
Financial impact	1.66 [1.00,2.76]	0.51	**0.05**	1.29 [0.91,1.81]	0.25	0.148
Someone close had COVID	1.83 [0.27,12.4]	0.6	0.537	6.4 [0.74,55.01]	1.86	0.09
Someone close was hospitalized	1.49 [0.21,10.6]	0.4	0.691	5.68 [0.65,49.77]	1.74	0.116
Someone close died	1.18 [0.17,8.14]	0.17	0.867	4.74 [0.55,40.88]	1.56	0.157
**Estimated COVID mortality**						
Underestimate	1.10 [0.56,2.17]	0.09	0.782	0.97 [0.56,2.17]	−0.03	0.915
Overestimate	1.34 [0.67,2.68]	0.29	0.415	1.34 [0.67,2.68]	0.29	0.188
**Likelihood of dying from COVID**						
High	2.91 [1.57,5.37]	−1.07	**<0.001**	2.3 [1.52,3.48]	−0.83	**<0.001**
Low	0.56 [0.22,1.45]	−0.58	0.230	0.61 [0.36,1.05]	−0.49	0.077
**COVID vaccine knowledge**						
Underestimate efficacy	13.9 [7.92,24.4]	2.63	**<0.001**	7.08 [4.85,10.35]	1.96	**<0.001**
Prior COVID diagnosis						
Recovered from COVID	1.88 [1.01,3.52]	0.63	**0.047**	2.58 [1.73,3.85]	0.95	**<0.001**
**Contact with COVID patients**						
Frequent	1 [0.71,1.44]	0.01	0.961	1.04 [0.82,1.33]	0.04	0.735
**Political party affiliation**						
Democratic	0.45 [0.22,0.95]	−0.79	**0.035**	0.47 [0.29,0.75]	−0.76	**0.002**
Republican	1.34 [0.72,2.47]	0.29	0.354	1.19 [0.77,1.83]	0.17	0.429
**Social media use**						
Well connected	1 [0.78,1.30]	0.01	0.977	0.85 [0.72,1.01]	−0.16	0.061
**News sources**						
Cable news	1.39 [0.67,2.87]	0.33	0.380	1.14 [0.69,1.89]	0.14	0.598
Mainstream news	1.77 [0.76,4.13]	0.57	0.184	1.29 [0.72,2.28]	0.25	0.392
Social media	0.50 [0.19,1.31]	−0.69	0.159	0.72 [0.39,1.32]	−0.33	0.287
Family or friends	0.69 [0.24,1.96]	−0.38	0.481	1.09 [0.55,2.20]	0.09	0.800

**Bold**—statistically significant predictors of vaccination intention.

**Table 5:** Characteristics of four clusters.

	Total (*n* = 304)	Group 1 (*n* = 38)	Group 2 (*n* = 94)	Group 3 (*n* = 86)	Group 4 (*n* = 86)
**Gender**					
Male	53 (17)	10 (27)	14 (15)	16 (19)	13 (15)
Female	251 (83)	28 (73)	80 (85)	70 (81)	73 (85)
**Age**					
1946–1964	50 (16)	16 (42)	18 (19)	9 (10)	7 (8)
1965–1980	87 (29)	10 (26)	29 (31)	28 (33)	20 (23)
1981–1996	155 (51)	11 (29)	46 (49)	46 (53)	52 (61)
After 1996	12 (4)	1 (3)	1 (1)	3 (4)	7 (8)
**Race**					
White	242 (80)	29 (77)	76 (81)	72 (84)	65 (75)
African American	27 (9)	2 (5)	8 (9)	6 (7)	11 (13)
Asian American	19 (6)	4 (10)	5 (5)	6 (7)	4 (5)
Pacific Islander	4 (1)	0 (0)	1 (1)	1 (1)	2 (2)
Native American	12 (4)	3 (8)	4 (4)	1 (1)	4 (5)
**Ethnicity**					
Hispanic	98 (32)	13 (34)	44 (47)	21 (24)	20 (23)
Non-Hispanic	206 (68)	25 (66)	50 (53)	65 (76)	66 (77)
**Education**					
Some college	40 (13)	3 (8)	28 (30)	7 (8)	2 (2)
Associate degree	56 (18)	4 (10)	28 (30)	16 (19)	9 (10)
Bachelor’s degree	128 (42)	15 (39)	31 (33)	46 (53)	36 (42)
Graduate degree	48 (16)	9 (24)	5 (5)	10 (12)	24 (28)
Doctoral degree	31 (10)	7 (18)	2 (2)	7 (8)	15 (17)
**Political Affiliation**					
Democratic	54 (18)	2 (5)	8 (9)	6 (7)	38 (44)
Republican	155 (51)	26 (68)	44 (47)	52 (60)	33 (38)
No lean	95 (31)	10 (27)	42 (44)	28 (33)	15 (18)
**Occupation**					
Physician	11 (4)	2 (5)	0 (0)	2 (2)	7 (8)
Nurse	144 (47)	20 (54)	31 (33)	49 (57)	44 (51)
NP/PA	5 (2)	2 (5)	0 (0)	1 (1)	2 (2)
Pharmacist	4 (1)	0 (0)	1 (1)	0 (0)	3 (3)
CRT/RRT	23 (7)	2 (5)	5 (5)	14 (16)	2 (2)
Administrator	11 (4)	5 (13)	1 (1)	3 (3)	2 (2)
Allied health	106 (35)	7 (18)	56 (60)	17 (20)	26 (30)
**Clinical Area**					
ICU	95 (32)	14 (37)	20 (21)	43 (51)	18 (21)
Non-ICU	116 (38)	10 (27)	41 (44)	25 (29)	40 (47)
Emergency room	25 (8)	5 (13)	2 (2)	9 (10)	9 (10)
Outpatient	68 (22)	9 (23)	31 (33)	9 (10)	19 (22)
**Willingness to receive COVID-19 vaccine**					
Definitely not	121 (40)	26 (69)	56 (60)	17 (20)	22 (26)
Probably not	102 (33)	12 (31)	31 (33)	27 (31)	32 (37)
Not sure	81 (27)	0 (0)	7 (7)	42 (49)	32 (37)
**Willingness to recommend COVID-19 vaccine**					
Definitely not	55 (18)	22 (58)	24 (25)	8 (9)	1 (1)
Probably not	98 (32)	14 (37)	41 (44)	25 (29)	18 (21)
Not sure	103 (39)	2 (5)	27 (29)	41 (48)	33 (38)
Probably yes	35 (11)	0 (0)	2 (2)	7 (8)	26 (31)
Definitely yes	13 (4)	0 (0)	0 (0)	5 (6)	8 (9)
**Knowledge of COVID-19 vaccine efficacy**					
Accurate	119 (39)	6 (16)	24 (25)	40 (47)	49 (57)
Underestimate	185 (61)	32 (84)	70 (75)	46 (53)	37 (43)
**Recent Flu vaccination receipt**					
Yes	195 (64)	12 (41)	62 (66)	53 (61)	68 (79)
No	109 (36)	26 (59)	32 (34)	33 (39)	18 (21)
**Perceived likelihood of dying from COVID-19**					
Low	261 (86)	33 (87)	90 (96)	63 (73)	75 (87)
Average	21 (7)	3 (8)	2 (2)	12 (14)	4 (5)
High	22 (7)	2 (5)	2 (2)	11 (13)	7 (8)
**Estimated mortality from COVID-19**					
Underestimate	137 (45)	29 (76)	55 (58)	31 (36)	22 (26)
Accurate	119 (39)	8 (21)	29 (31)	38 (44)	44 (51)
High	48 (16)	1 (3)	10 (11)	17 (20)	20 (23)
**Seasonal flu is more contagious than COVID-19**					
Yes	68 (22)	30 (79)	18 (19)	12 (14)	8 (9)
No	93 (31)	2 (5)	19 (20)	31 (36)	41 (48)
Not sure	142 (47)	6 (16)	57 (61)	43 (50)	37 (43)
**Seasonal flu is deadlier than COVID-19**					
Yes	48 (16)	24 (63)	15 (16)	5 (6)	4 (5)
No	136 (45)	4 (10)	19 (20)	66 (77)	47 (55)
Not sure	120 (39)	10 (26)	60 (64)	15 (17)	35 (40)
**COVID-19 is a manmade virus**					
Yes	108 (35)	35 (92)	40 (42)	22 (25)	11 (13)
No	94 (31)	2 (5)	17 (18)	13 (15)	62 (72)
Not sure	102 (34)	1 (3)	37 (40)	51 (60)	13 (15)
**COVID-19 is a hoax**					
Yes	32 (10)	27 (71)	2 (2)	2 (2)	1 (1)
No	238 (78)	5 (13)	69 (73)	79 (92)	85 (99)
Not sure	33 (11)	6 (16)	22 (25)	5 (6)	0 (0)
**The impact of COVID-19 is exaggerated**					
Yes	104 (33)	34 (89)	50 (53)	11 (13)	9 (10)
No	153 (50)	1 (3)	24 (25)	60 (70)	68 (80)
Not sure	47 (15)	3 (8)	20 (22)	15 (17)	9 (10)

## Data Availability

The data that support the findings of this study are not openly available due to institutional policies and are available from the corresponding author upon reasonable request in a controlled access.
